# Effects of Partially Ionised Medical Oxygen, Especially with O_2_•^−^, in Vibration White Finger Patients

**DOI:** 10.3390/ijerph110605698

**Published:** 2014-05-27

**Authors:** Slavomír Perečinský, Lenka Murínová, Ivan Engler, Viliam Donič, Pavol Murín, Marek Varga, Ľubomír Legáth

**Affiliations:** 1 Department of Occupational Medicine and Clinical Toxicology, Faculty of Medicine, Pavol Jozef Safarik University, 04190 Kosice, Slovak Republic; E-Mails: slavomir.perecinsky@upjs.sk (S.P.); murinovalenka@yahoo.com (L.M.); marek.varga@upjs.sk (M.V.); 2 Department of Occupational Medicine and Clinical Toxicology, Louis Pasteur University Hospital, 04190 Kosice, Slovak Republic; 3 Department of Human Physiology, Faculty of Medicine, Pavol Jozef Safarik University, 04001 Kosice, Slovak Republic; E-Mails: engler@io2th.com (I.E.); viliam.donic@upjs.sk (V.D.); 4 Cardiology Clinic, Faculty of Medicine, Pavol Jozef Safarik University, 04001 Košice, Slovak Republic; E-Mail: murinpavol@yahoo.com; 5 Cardiology Clinic, East Slovakian Institute of Cardiovascular Diseases, 04001 Košice, Slovak Republic

**Keywords:** hand-arm vibration syndrome, vibration white finger, cold provocation test, ionized oxygen therapy, O_2_•^−^, O_2_, finger plethysmography

## Abstract

A major symptom of hand-arm vibration syndrome is a secondary Raynaud’s phenomenon—vibration white finger (VWF)—which results from a vasospasm of the digital arteries caused by work with vibration devices leading to occupational disease. Pharmacotherapy of VWF is often ineffective or has adverse effects. The aim of this work was to verify the influence of inhalation of partially ionized oxygen (O_2_•^−^) on peripheral blood vessels in the hands of patients with VWF. Ninety one (91)patients with VWF underwent four-finger adsorption plethysmography, and the pulse wave amplitude was recorded expressed in numeric parameters—called the native record. Next, a cold water test was conducted following with second plethysmography. The patients were divided in to the three groups. First and second inhaled 20-min of ionized oxygen O_2_•^−^ or oxygen O_2_ respectively. Thirth group was control without treatment. All three groups a follow-up third plethysmography—the post-therapy record. Changes in the pulse wave amplitudes were evaluated. Inpatients group inhaling O_2_•^−^ a modest increase of pulse wave amplitude was observed compared to the native record; patients inhaling medical oxygen O_2_ and the control showed a undesirable decline of pulse wave amplitude in VWF fingers. Strong vasodilatation were more frequent in the group inhaling O_2_•^−^ compare to O_2_ (*p* < 0.05). Peripheral vasodilatation achieved by inhalation of O_2_•^−^ could be used for VWF treatment without undesirable side effect in hospital as well as at home environment.

## 1. Introduction

Hand-arm vibration syndrome (HAVS) is a group of illnesses including damaged blood vessels, nerves, muscles, bones and joints of the upper limbs, arising as the local effect of excessive vibration in the upper limbs [1,2,3]. A secondary Raynaud’s phenomenon, also known as vibration white finger (VWF), is among the most significant symptoms. This arises on the basis of a vasospasm of the digital arteries or arteries in the palmar arch, most often initiated by cold [4]. A characteristic manifestation of ischemia is a temporary paleness of the fingers often associated with insensibility and paresthesia of the afflicted fingers. Even though the pathogenesis of the illness is not completely clear, it is assumed that the mechanism of the vasospasm is central and local. The central mechanism may be caused by an increased activity of the sympathetic nervous system and a local disturbance of the blood vessels [5,6]. Local effect of vibrations and cold increases the activity of the sympathetic nervous fibers supplying the tiny arteries in the fingers. Vibrations directly damage the artery wall and influence the secretion activities of the endothelium. This leads to the production of the vasoconstricting endothelin, thrombomodulin and other substances influencing the proliferation processes and the aggregation of thrombocytes. The pathological process can in the final phase result in morphological changes proliferation of media of the small arteries or arterioles, fibrosis in the arterial wall and the perivascular space, microangiopathy with capillary bleeding and micropetechiae [7,8,9]. Millions of workers in many professions around the world are exposed to vibrations and are at risk of the occurrence of VWF. The local effects of vibrations occur when working with pneumatic jack hammers, grinders and chainsaws. The prevalence of VWF in these professions can be very high, even more than 71%; however, this varies significantly depending on the type and duration of exposure to vibration and the climatic conditions [3,10,11]. In many industrialized countries VWF ranks among the most commonly reported occupational diseases. In some cases a detailed case history is sufficient for diagnosing the disease [5]; in the case of occupational disease, however, objective diagnostic methods are essential. Even though no unified diagnostic method which could be considered as a gold standard exists at present, the most commonly used method is the so-called cold test, with immersion of the hands into cold water. The subsequent objectivization of the vasospasm is commonly performed using a finger plethysmography. During this examination a record of a plethysmographic curve documenting the local engorgement is obtained, and the shape and dicrotic character of the curve is assessed, as is the change in the shape and the distribution of the pulse curve. With complete occlusion of the digital artery by a vasospasm, the disintegration of the curve occurs; at this point it is no longer possible to detect its physiological shape and course [12]. A therapy for VWF is not essential in many cases and simply eliminating exposure, working in a warm environment and stopping smoking, which worsens the disease, suffices [13]. In more difficult stages, however, treatment is indicated. At present the first choice is the use of calcium channel blockers of the dihydropyridine type, and with insufficient effect or intolerance ACE inhibitors, α-blockers, nitrates or selective inhibitors of reverse capturing of serotonin are indicated. This vasodilatation treatment is, however, often ineffective or has many adverse effects. The occurrence of hypotension, flushing, dizziness and peripheral edema is typical undesirable side effects [14,15]. The beneficial effect could be expected in inhalation of partially ionized medical oxygen. It may interfere with NO vasodilatation mechanisms and serotonin balance. Partially ionized oxygen (O_2_•^−^) can be produced by ionization of medical oxygen (O_2_) in the high-voltage chamber of the Oxygen Ion 3000/Dr. Engler device [16]. Partially ionized oxygen (O_2_•^−^) compared to medical oxygen (O_2_), showed many different physiological effects [17]. The device has been commercially available since 2000 and is produced by CS Tronic (St Pantaleon, Austria) as a medical device with EU certificate. It is commonly used for treatment of bronchial asthma. The therapeutic effect of ionized waterfall aerosol on pediatric allergic asthma was demonstrated [18]. According to our knowledge, the effect of inhalation of ionized oxygen on the peripheral vascular flow has been not evaluated in human subject yet.

Therefore, the aim of our study was to verify the possible vasodilatation effect of inhalation of ionized oxygen (O_2_•^−^) on the blood vessels of the fingers in patients with VWF and to compare it with the effect of inhalation of medical oxygen (O_2_) and with control.

## 2. Material and Methods

### 2.1. Selection of Subjects

In the period from 2011–2012 a total of 335 patients were examined at our Department of Occupational Medicine and Clinical Toxicology. The patient’s case histories presented color changes to the fingers as in blanching or cyanosis accompanied by pain and paresthesia, provoked by cold. A cold test was performed on all patients followed by objective measurement a finger plethysmography. A total of 91 patients in whom the complete disintegration of the pulse waves in at least one finger was recorded using plethysmography were included in the study. 

Only patients who currently or in the past were exposed to vibrations in the work environment were included. Patients who in spite of a case history of symptoms exhibited no complete disintegration of the pulse waves were excluded, as were patients in whom disintegration of the pulse waves had occurred prior to the cold test. Because of the possible impact of the season of the year on the plethysmography, only patients who were examined in the months of November to April were analyzed. 

We analyzed the number of measurement (fingers) rather than patients and it could appear as the limitation of the study. The reason was that fingers are affected individually by VWF. On the same hand appeared healthy fingers and fingers with different degree of disease. For us were useful only fingers with VWF. Healthy fingers were excluded from analysis.

All subjects gave their informed consent for inclusion before they participated in the study. The study was conducted in accordance with the Declaration of Helsinki, and was approved by the Ethics Committee of Faculty of Medicne, Pavol Jozef Safarik University (EC: No. 23/1/2012). 

### 2.2. Investigational Methods

The tests were performed in the morning hours. Patients were warned not to use pharmaceuticals having a vasodilatation effect before the test. They likewise were asked to not drink warm drinks, especially tea and coffee, and not smoke. Before the start of the test, they were acclimatized to the laboratory temperature of 21–24 °C for a minimum of 10–15 min. After the acclimatization period a four-finger adsorption plethysmography (Pulsatrix 40, Technicare CZ, Ostrava, Czech Republic) was performed with the recording of the amplitudes of the pulse wave expressed also in a numeric parameters. This first plethysmography was called the native record. The plethysmographic receptors were placed at the tip of each four fingers (2nd to 5th) of both hands. Emitted infrared light is reflected by erythrocytes. The photo detector detects the reflected light and converts it into an electrical signal, which is recorded in the form of a plethysmographic curve. The curve disintegrates in the case of transient occlusion of the digital artery after cooling, so it is impossible to detect its physiological shape and course. The cold test was conducted by the standard method according to Rejsek [19]. A patient in a sitting position immersed the upper limb above the elbow in cold circulating water of temperature 12‒15 °C for 10 min. Immediately after pulling the arms from the water a second plethysmographic examination was performed. This was called record after cooling. For evaluation of the curve, the decline of the amplitude of the pulse wave was expressed as a percentage. The decline of the amplitude of the pulse wave by 99% and more compare to the native record represent a complete disintegration of the curve which means the transient occlusion of digital blood flow.Immediately after second plethysmograpy the patients was divided in to the three homogenous groups according to the severity of finger VWF. The first Group 1 (n = 26) started a short-term 20-min inhalation of partially ionized oxygen (O_2_•^−^ 200,000 ions/ccm at a flow 8 L/min, Oxygen Ion 3,000/Dr. Engler). The second Group 2 (n = 27) inhaled 20-min medical oxygen (O_2_ flow 8L/min). The third Group 3 (n = 38) was a control group rest without any described treatment. All patients were left in quiet room at 20 °C temperature. After 20 min of inhalation, or after remaining at rest in the control group, a third plethysmographic was performed called the post-therapy record. The change in the amplitude of the pulse waves in comparison with the native record, evaluated as a percentage, was assessed. The investigator who performed cold provocation test and plethysmography was blinded to the which group belong the subjects. 

In order to avoid repeating the cooling test three times on the same patient we divided patients into three groups. Each patient underwent the cooling test only once. However, all groups of patients were not different in age and severity of Raynaud phenomena, which mean all groups were practically homogenous and identical. Therefore impact of this point on the obtained results was minimized.

### 2.3. Statistical Analysis

Statistical analysis was performed in Arcus Quickstat Biomedical. The results were expressed as the average ±SD. For comparing the differences between the groups of patients, an analysis of variance (ANOVA) was used with subsequent use of the Tukey-Kramer test (the age of patients). Data which did not show normal distribution were analyzed using the Kruskal-Wallis test (the number of disintegrations of pulse waves in individual patients, changes in the amplitude of the pulse waves). The absolute numbers of fingers with VWF in individual groups by category of differences of pulse waves were compared using the Chi-squared test, than it was graphically expressed in percentage. 

## 3. Results

The sample comprised 91 patients with transient occlusion of digital blood flow after cooling—88 men and three women. The number of patients and the average age of the patients and the number of disintegrations of pulse waves on fingers showing VWF (*i.e.*, the number of measurements) in the each group are presented in Table 1. The patient’s age differences between the groups were not significant. Likewise, at native record no statistically significant difference in the average number of disintegration of pulse waves in one patient was found. This mean the all three groups were homogenous.

**Table 1:** Characteristics of the three groups.

Group	Number of Patients	Average Age	Average Number of Disintegrations	Number of Fingers with VWF (Measurements)
1. O_2_•^−^	26	51.04 (±7.07)	2.78 (±2.30)	64
2. O_2_	27	54.65 (±8.28)	2.82 (±2.06)	65
3. Control	38	53.03 (±8.64)	2.96 (±2.37)	89

The assessment of the change of amplitude in the pulse waves in Group 1 patients inhaling O_2_•^−^ showed a mild increase of pulse wave amplitude in the post-therapy record compare with the native record (+0.68%). In group 2 O_2_ caused an undesirable decline in the amplitude of the pulse wave (Table 2). Using the Kruskall-Wallis test, a statistically significant difference was found between the group of patients inhaling negative ionized oxygen O_2_•^−^ compare to control group (*p* < 0.01) and compare to medical oxygen group (*p* = 0.018). There was not statistical significant difference between control and medical oxygene group.

**Table 2:** Differences between the VWF amplitude of the pulse wave measured after 10 min cooling following by 20 min of O_2_•^−^ or O_2_ respectively inhalation (except control) and native record.

Group	Difference in the Amplitude of the Pulse Wave
1. O_2_•^−^	+0.68% (±51.39)
2. O_2_	−20.54% (±44.36)
3. Control	−24.44% (±50.79)

Based on differences in the pulse wave amplitudes between the native record and the post-therapy record, the values of the increases in the pulse wave amplitudes were divided into three categories: (1) “no vasodilatation” (a difference of amplitude from −100% to −90%); (2) “vasodilatation” (a difference of amplitude −89% to 0%); (3) “strong vasodilatation”, where the post-therapy amplitude was higher than the amplitude with the native record (a difference of amplitude >0%).

*Category 1 “No vasodilatation”*

No increase (or only a minor increase) in the amplitude of the pulse wave was observed in 19 measurements—fingers (from all 218 VWF). In the control group this represented 12% of all measurements, in the group inhaling O_2_•^−^ only 5% and in the group inhaling oxygen O_2_ 6%. This category represents most severely damaged fingers, probably vasoparalytic.

*Category 2 “Vasodilatation”*

In the majority of number of measurements (fingers) the differences in the amplitude of the pulse waves were found to be in the range from ‒89% to 0%. No significant difference between groups were found. 

*Category 3 “Strong vasodilatation”*

**Figure 1:**
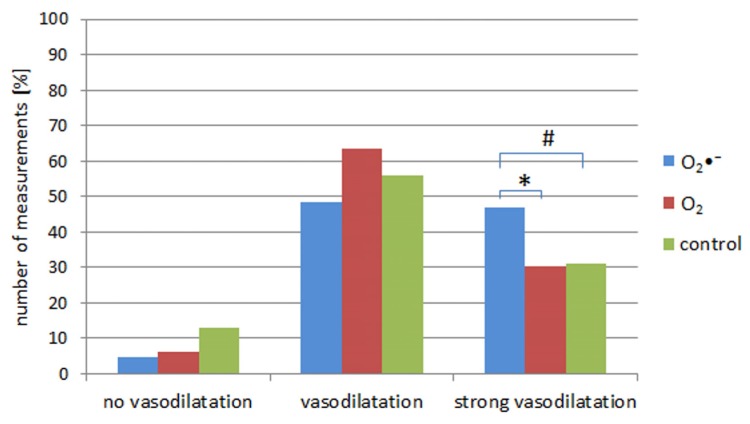
Present three categories fingers with VWS showing the respond to inhalation of O_2_•^−^ or O_2_ compared to control: (1) category “no vasodilatation”; (2) “vasodilatation”; (3) “strong vasodilatation”. The column expresses a number of measurements (it means number of fingers with desintegration of pulse waves) in percentage on the basis of an increase in the amplitude of the pulse wave. It is a marker of dilatation effect on peripheral blood vessels on the fingers.

Notes: * *p* < 0.05, **# ***p* = 0.065.

The biggest differences in the number of measurements (fingers) amplitude of the pulse wave was observed in category “strong dilatation” (>0%). Inhalation of O_2_•^−^ showed a significant vasodilatation 47% compared to group 2 with O_2_ inhalation 30% (*p* < 0.05) and borderline significant difference compared to group 3—control 31% (*p* = 0.065). No significant difference was shown between group 2 with O_2_ inhalation and control group 3 (Figure 1).

## 4. Discussion

In 1980 in Salzburg, Austrian surgeon I. Engler introduced Ionised Oxygen Therapy (IO_2_Th). This method including inhalation of medical oxygen O_2_ enriched with oxygen O_2_•^−^ or O_2_•^+^ ions produced by a special device. It was shown that IO_2_Th with O_2_•^−^ decreases serotonin [20], acts vagotonically, increases physical performance in the humans. On the other hand O_2_•^+^ increases serotonin level [20], tonus of sympathicus and acts as a scavenger of free radicals. Inhalation of ionized oxygen has showed no undesirable side effects. Therefore it is using for treatment of bronchial asthma in children [16]. For this reasons a beneficial effect of O_2_^•-^ inhalation in patients with vibration white fingers (VWF) was expected. 

For diagnostics of VWF as an occupational disease in the clinical practice a cold test combined with finger plethysmography is routinely used. This simple, non-invasive and objective method, easy to perform in patients with VWF, represents a useful model for the study of the biological effects of ionized oxygen. The advantage—the study can be carried out as a part of standard diagnostic procedure. Patients have no problems to participate. They do so voluntarily and the procedure does not bother them. Because only a standard and approved medical method was used, ethics committee consent was not required in this case. 

In our work we observed an increase in the amplitude of the pulse wave compared to measuring after cooling in the majority of measurements in all groups, since the impact of the increased temperature of the environment in comparison with the temperature of the water during the cold test led to vasodilatation. However, the strong vasodilatation, *i.e.*, the higher amplitude of the post-therapy pulse wave in comparison with the native record, was obtained in only one-third of cases, and most of them were in the group inhaling O_2_•^−^. 

In 8.52% of cases, the vasospasm persisted even 20 min after the end of the cold test. The majority of these measurements belonged to the control group; in contrast the lowest number of vasospasm was in the group inhaling O_2_•^−^. 

The increase in the amplitude of the pulse wave after therapy was obtained in all groups of patients; however, there was a statistically significant difference between the group inhaling O_2_•^−^ compared to the other groups. 

In the control group only the time factor caused vasodilatation. Similar results were also obtained in the group of patients inhaling medical oxygen O_2_. The results of our work confirm that for vasodilatation, aside from the time factor inhalation of ionized oxygen O_2_•^−^ has an additional beneficial dilatation effect. 

During the cold test, the activity of the sympathetic system increases, and it participates in the origin of a vasospasm of the digital arteries. Inhalation of ionized oxygen (O_2_•^−^), with its vagotonic effect probably inhibit these adrenergic stimuli [16]. Aside from increased sympathetic activity, endothelium dysfunction also takes part in the symptoms of VWF. It leads to an imbalance between the production of vasodilatation and vasoconstriction factors, with vasoconstriction effects on the endothelium. 

It is probably possible that O_2_•^−^ increases the NO production according to the reaction N_2_ + O_2_•^−^ = 2NO [16]. The reduced production of NO in patients with VWF is the reason for the absence of reflexive vasodilatation after cooling, with persist vasospasm. This can also occur in a portion of the healthy population, however, it lasts a maximum 2 min after cooling. In patients with VWF it persists even 15 min after cooling with a gradual reperfusion of the fingers [21]. The spontaneous loosening of the vasospasm can explain the spontaneous vasodilatation effect observed in all three groups. 

Post-therapy strong vasodilatation associated with increasing of the amplitude of the pulse wave exceeding the level of the native record amplitude, was found more frequently in the group inhaling O_2_•^−^. This finding confirms a significant vasodilatation effect of O_2_•^−^ on peripheral circulation. 

A favorable effect of O_2_•^−^ can also be explain by decreasing of serotonin production. Inhibition of inflammatory mechanisms may have beneficial effect in patients with VWF [20,22]. It is known that medical oxygen (O_2_) during neurosurgical operations causes vasospasms in brain blood vessels. 

Gaseous negative ions induce stimulation of cytochrome biosynthesis [17]. ATP is, however, not only an energetic but also an informational molecule for vascular tonus in regard to dilatation or spasms [23]. Traces of O_2_•^−^ in medical oxygen also seem to affect the production of energetic ATP and decrease the level of lactate [16]. 

O_2_•^−^, is consider the strongest radical, so-called reactive oxygen species (ROS), which escalation can cause many chronic diseases. According to the hormesis theory, high doses of radon respectively. of radicals can cause cancer, while lower doses may have anti-radical effect [24]. In our study we used O_2_•^−^ 200,000 ions/ccm O_2_ which represents a picodosis.

## 5. Conclusion

The results of this study showed the beneficial vasodilatation effect of a single inhalation of ionized medical oxygen (O_2_•^−^) lasting 20 min on the peripheral vascular flow in patients with VWF compared to inhalation of O_2_ and to control. This simple biophysical method, without side-effects, can be use for home or in hospital therapy. It can be use in VWF as an alternative to the pharmacological treatment, which is poorly tolerated by patients due to the undesirable side-effects. 
